# Identifying and Managing Areas under Threat in the Iberian Peninsula: An Invasion Risk Atlas for Non-Native Aquatic Plant Species as a Potential Tool

**DOI:** 10.3390/plants12173069

**Published:** 2023-08-26

**Authors:** Argantonio Rodríguez-Merino

**Affiliations:** Independent Researcher, 28019 Madrid, Spain; argantonio.rodriguez@gmail.com

**Keywords:** alien species, biological invasions, MaxEnt, non-native species, species distribution models, virtual atlas

## Abstract

Predicting the likelihood that non-native species will be introduced into new areas remains one of conservation’s greatest challenges and, consequently, it is necessary to adopt adequate management measures to mitigate the effects of future biological invasions. At present, not much information is available on the areas in which non-native aquatic plant species could establish themselves in the Iberian Peninsula. Species distribution models were used to predict the potential invasion risk of (1) non-native aquatic plant species already established in the peninsula (32 species) and (2) those with the potential to invade the peninsula (40 species). The results revealed that the Iberian Peninsula contains a number of areas capable of hosting non-native aquatic plant species. Areas under anthropogenic pressure are at the greatest risk of invasion, and the variable most related to invasion risk is temperature. The results of this work were used to create the Invasion Risk Atlas for Alien Aquatic Plants in the Iberian Peninsula, a novel online resource that provides information about the potential distribution of non-native aquatic plant species. The atlas and this article are intended to serve as reference tools for the development of public policies, management regimes, and control strategies aimed at the prevention, mitigation, and eradication of non-native aquatic plant species.

## 1. Introduction

When non-native species arrive and become established in new areas, there are often environmental and/or economic costs associated with the impacts of the species and the resulting management needs [1,2,3]. In addition, biological invasions are considered a major threat to natural systems and a key cause of biodiversity loss worldwide [4,5]. They are a problem that shows no signs of abating, given that the number of non-native species has not stopped growing in recent decades and is expected to continue along this global trajectory for the foreseeable future [6,7,8].

Consequently, predicting the risk of biological invasions remains one of the greatest challenges in conservation [9] and, therefore, it is essential to develop mitigation strategies that target future invasions [10]. In addition, gathering information on invasion risk is a fundamental part of horizon scanning, a technique that can guide management strategies for eradicating or controlling populations of established non-native species and preventing the arrival and establishment of non-native species with invasive potential [11]. Early warning systems can play an extremely important role, given that control and eradication are generally most effective when efforts are deployed during the introduction phase of the invasion before species become established [9].

Species distribution models (SDMs) are currently the most common approach used to explain and predict where species occur in space and time [12]. Using species occurrence data and a set of predictor variables, SDMs can infer species distributions in areas for which no observations are available [13]. They are thus helpful tools for identifying the areas most likely to be colonized by and host a given non-native species; in this way, it is possible to determine which areas are at the greatest risk of invasion [14].

Inland aquatic ecosystems, both freshwater and estuarine, provide innumerable goods and services and are, at the same time, especially vulnerable to biological invasions compared to terrestrial ecosystems for three key reasons. First, they experience a high rate of species introductions because of human activities. Second, they tend to be quite isolated. Third, they host a large number of endemic species [11,15,16,17]. Some of the most harmful invaders in inland aquatic ecosystems are aquatic plant species, given their ability to alter habitat structure and composition as well as ecosystem biogeochemistry and water quality [18]. Research suggests that the number of non-native aquatic plants that have been introduced to the Iberian Peninsula has been increasing over recent years [19], a pattern also observed in Europe [20]. Such is clearly concerning for inland aquatic habitats and their native species, especially considering that the Iberian Peninsula is particularly species-rich and harbors a large number of endemic taxa [21]. Yet, few resources are currently available for monitoring non-native species, especially inconspicuous groups such as aquatic plant species, which limits knowledge of the potential distribution of invasive species and the ability to develop appropriate preventive measures. In this context, the SDMs could be used to help governmental authorities direct time, resources, and energy to the locations at greatest risk of invasion. Indeed, for the Iberian Peninsula, SDMs have been successfully used with both established non-native aquatic plants [19,20] and with non-native aquatic plant species with invasive potential [22].

Although information on the occurrence and global distribution of non-native species is becoming increasingly available, thanks to databases and platforms such as GBIF (https://www.gbif.org/, accessed on July 2023), it remains difficult to obtain information on the potential distribution and hence invasion risk for certain species. This challenge means that environmental managers and planners lack the full breadth of data they need to develop and implement effective management and conservation strategies.

This study thus aims to fill some of the information gaps that exist regarding the potential distribution of non-native aquatic plant species in the Iberian Peninsula and to create practical resources from this information. The main objective is to produce a map of suitable areas for assessing the invasion risk of invasive and potentially invasive species in the Iberian Peninsula. To achieve this, there are three specific objectives: (1) to establish the methodological basis for mapping potential species distributions and, consequently, determining the invasive potential of non-native aquatic plants; (2) to identify the areas at greatest risk of invasion by non-native aquatic plants; and (3) to characterize these areas based on climatic, topographical, and anthropogenic variables.

## 2. Results

### 2.1. Occurrence Data

This study looked at 72 target species: 32 non-native species that have already become established in the Iberian Peninsula and 40 non-native species with invasive potential. After filtering the original data used to create the models, the record number ranged between 68 and 15,072 for the established species (Table S1) and between 22 and 6212 for the species with invasive potential (Table S2). The decision was made to perform the models without *Lemna aequinoctialis* Welw., a species that had been on the list created by the LIFE INVASAQUA Project (LIFE17 GIE/ES/000515) (hereafter Life Invasaqua project) [23]. Although good results were obtained with few data, *L. aequinoctialis* had too few records of occurrence (*n* = 3) once the protocol for cleaning up the distribution data had been applied.

### 2.2. Distribution of Currently Established Non-Native Aquatic Plant Species

**Figure 1:**
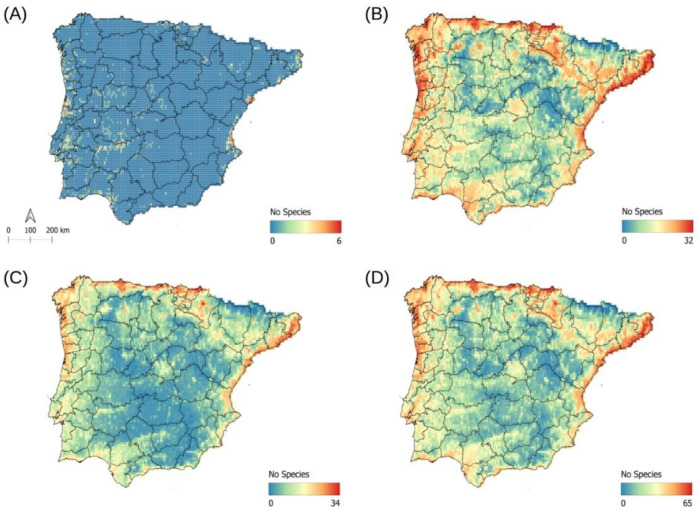
(**A**) Map of observed species richness for non-native aquatic plant species; (**B**) Map of potential species richness for established non-native aquatic plant species; (**C**) Map of potential species richness for non-native aquatic plant species with invasive potential; (**D**) Map of potential species richness for both established species and species with invasive potential.

The current known distribution of non-native aquatic plant species in the Iberian Peninsula are represented in Figure 1A. Of the 9039 cells in the regional map, 759 cells contained instances of occurrence for non-native aquatic plants. A given cell was occupied by one to six species (1 species: 555 cells; 2 species: 126 cells; 3 species: 40 cells; 4 species: 17 cells; 5 species: 19 cells; and 6 species: 2 cells). In general, most occupied cells were found in the western half of the Iberian Peninsula, specifically in coastal Portugal, around the mouth of the Tagus River, across a portion of the Guadiana Basin, and the coast of the southwestern Iberian Peninsula. Additionally, cells along the coast of the eastern Peninsula were occupied by a large number of non-native aquatic plant species. Indeed, only two cells containing six different species of non-native aquatic plants were found in this area.

### 2.3. Species Distribution Models

Overall, the models yielded highly accurate predictions. For the established species, AUC values ranged from 0.831 to 0.993 (mean AUC ± SD: 0.918 ± 0.058) (Table S1). For the species with invasive potential, the range was 0.810–0.993 (mean AUC ± SD: 0.940 ± 0.043) (Table S2). The lowest AUC value of 0.702 was associated with an established species, *Elodea canadensis* Michx.

**Figure 2:**
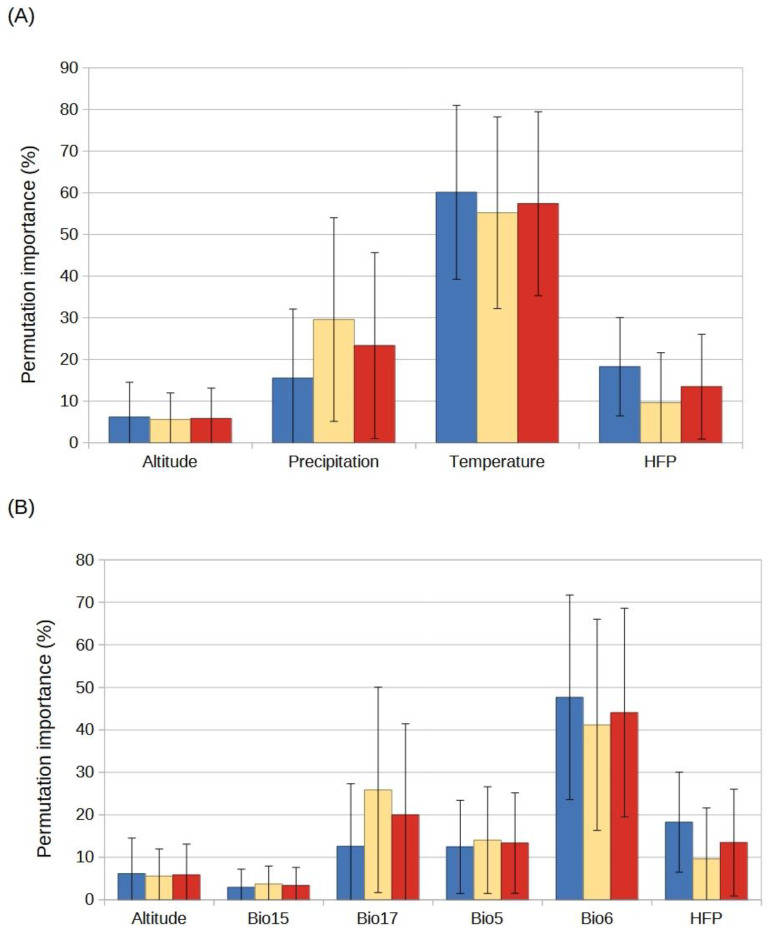
Relative importance (%) of the predictor variables used during SDM development and the standard error for each grouping of the target species: (**A**) Main variable types and (**B**) Independent variables. Bio5 = maximum temperature of the warmest month. Bio6 = minimum temperature of the coldest month. Bio15 = seasonality of precipitation. Bio17 = precipitation of the driest quarter. HFP = human footprint index. Altitude = digital elevation model. Blue bars: established species, yellow bars: potential species, and red bars: all established and potential species.

**Figure 3:**
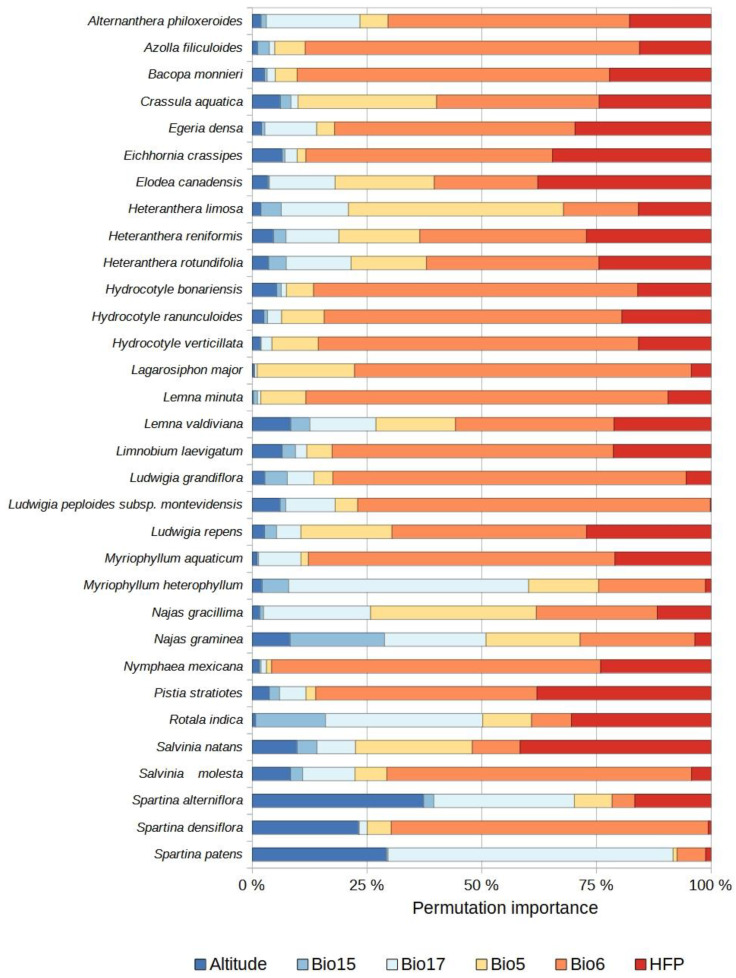
Relative importance (%) of the variables used to model the potential distributions of each established non-native aquatic plant species. Bio5 = maximum temperature of the warmest month. Bio6 = minimum temperature of the coldest month. Bio15 = seasonality of precipitation. Bio17 = precipitation of the driest quarter. HFP = human footprint index. Altitude = digital elevation model.

**Figure 4:**
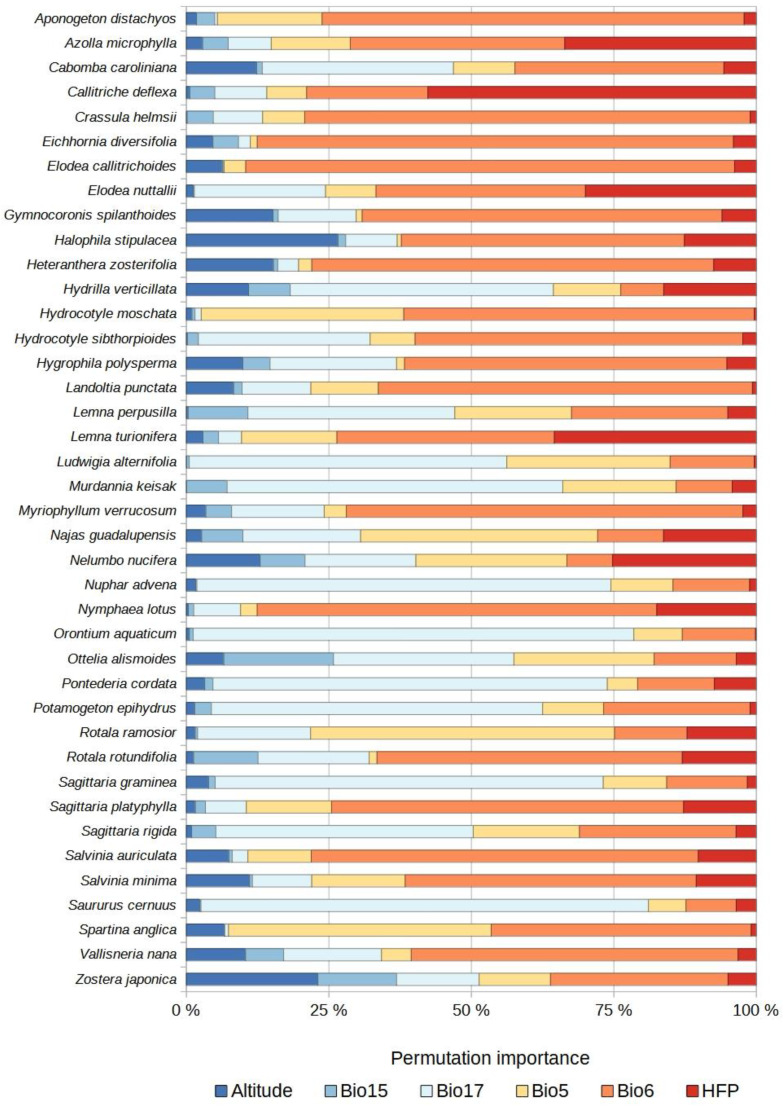
Relative importance (%) of the variables used to model the potential distributions of each non-native aquatic plant species with invasive potential. Bio5 = maximum temperature of the warmest month. Bio6 = minimum temperature of the coldest month. Bio15 = seasonality of precipitation. Bio17 = precipitation of the driest quarter. HFP = human footprint index. Altitude = digital elevation model.

For the established species, the most important predictor variables were temperature (combination of Bio5 and Bio6) as well as HFP and precipitation (combination of Bio15 and Bio17) (Figure 2A). For the species with invasive potential, these variables were temperature, precipitation, and HFP, the same variables as for the two groups of species combined (Figure 2A). Altitude was the variable of least importance for the established species, the species with invasive potential, and both groups combined (Figure 2A). For the established species, variable importance independently was ranked as follows: Bio6 > HFP > Bio17 > Bio5 > Altitude > Bio15 (Figure 2B and Figure 3). For the species with invasive potential, the order of ranking was: Bio6 > Bio17 > Bio5 > HFP > Altitude > Bio15 (Figure 2B and Figure 4). For both groups combined, it was: Bio6 > Bio17 > HFP > Bio5 > Altitude > Bio15 (Figure 2B).

### 2.4. Potential Species Distributions (Potential Invasion Risk)

**Figure 5:**
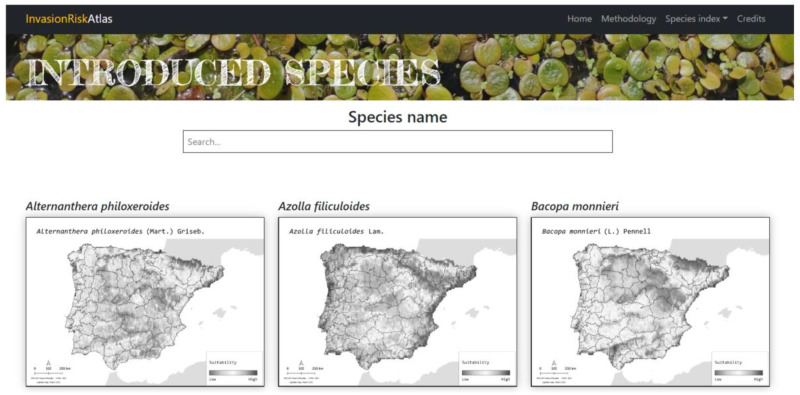
Illustration of the interface for the online Invasion Risk Atlas (https://InvasionRiskAtlas.github.io/, accessed on July 2023).

**Figure 6:**
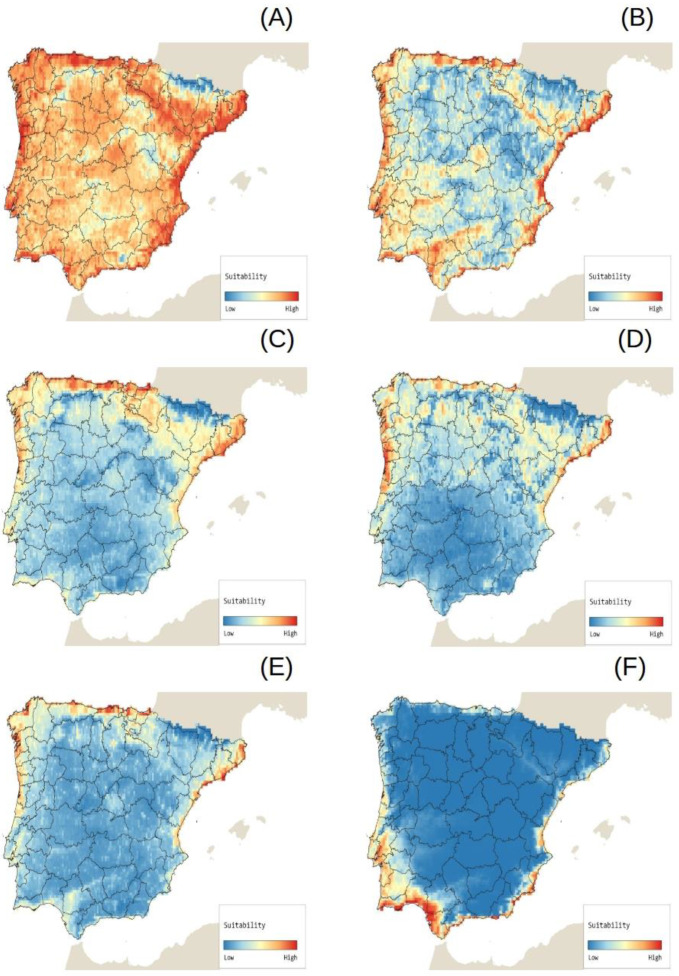
Potential distributions (invasion risk maps) of six non-native aquatic plant species that have been identified by experts as very high risk for Iberian inland waters (Life Invasaqua project; [23]): (**A**) *Azolla filiculoides* Lam. [24]; (**B**) *Eichhornia crassipes* (Mart.) Solms [25]; (**C**) *Ludwigia grandiflora* (Michx.) Greuter & Burdet [26]; (**D**) *Salvinia natans* (L.) All. [27]; (**E**) *Salvinia molesta* D.S. Mitch. [28]; (**F**) *Spartina densiflora* Brongn. [29].

The potential distribution of each target species is included in the online Invasion Risk Atlas ([24], Figure 5). Here zones and macroecological patterns associated with the greatest risk of invasion by each target species are indicated, whose distributions vary across the Iberian Peninsula (Figure 6).

### 2.5. Potential Richness of Non-Native Aquatic Plant Species

For the established species, the species with invasive potential, and both groups combined, potential species richness displayed similar associations with macroecological patterns (Figure 1B–D). Although the risk of invasion was heightened for the established species versus the species with invasive potential, the areas at greatest risk coincided: with coastal areas, large river basins, and densely populated urban centers. At the lowest risk were the most isolated zones of the Iberian Peninsula, namely high-elevation areas and areas far from large river basins and urban centers (Figure 1B–D).

**Figure 7:**
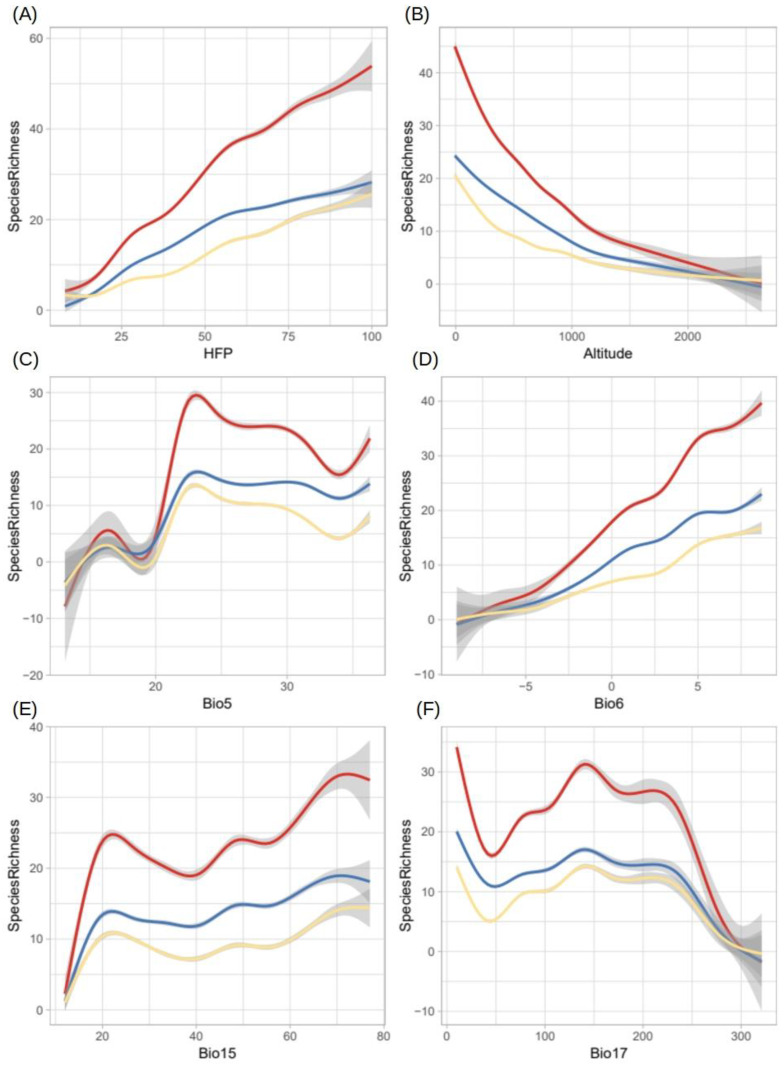
Response curves showing the relationship between the potential species richness of non-native aquatic plant species and the variables used in SDM development. The data are for the Iberian Peninsula. The solid lines represent the mean (blue lines: established species, yellow lines: potential species, and red lines: all established and potential species), and the shaded areas represent the 95% confidence intervals. (**A**) HFP index; (**B**) Digital elevation model (Altitude); (**C**) Maximum temperature of the warmest month (Bio5); (**D**) Minimum temperature of the coldest month (Bio6); (**E**) Seasonality of precipitation (Bio15); (**F**) Precipitation of the driest quarter (Bio17).

The established species, the species with invasive potential, and both groups together showed similar responses to the variables used in the SDMs in terms of areas with the highest potential species richness (Figure 7A–F). HFP was positively correlated with potential species richness: the areas experiencing more anthropogenic pressure seem to be at greater risk of invasion by the target species (Figure 7A). Lower elevation areas appear to be more likely to host a larger number of non-native aquatic plant species (Figure 7B). With regards to temperature, species richness increased with Bio6 (Figure 7D) and was higher for Bio5 at values between 20 °C and 25 °C (Figure 7C). There was also a relationship between precipitation and potential species richness. First, areas with greater seasonality in precipitation seem more likely to be invaded by a greater number of species (Figure 7E). Precipitation levels displayed a bimodal association: potential species richness was high in areas with low precipitation (<20 mm) and areas with higher precipitation (100–220 mm); between and above these precipitation levels, potential species richness decreased drastically (Figure 7F).

## 3. Discussion

Identifying and understanding the distribution of non-native species is one of the keys to establishing appropriate management strategies aimed at preventing, mitigating, and eradicating invasions. The Iberian Peninsula already hosts a large number of non-native aquatic species and is at substantial risk of future invasions [23]. This study underscores that lack of knowledge about the potential distribution of non-native aquatic plant species is one of the greatest limitations in biological invasion management and, consequently, about the possible threat they pose to the Iberian Peninsula, an important hotspot for aquatic plant species in Europe and around the Mediterranean [30,31].

Although information exists on the distribution of non-native plant species in the Iberian Peninsula, most are scattered across different sources, such as digital platforms and atlases. It also tends to be very general in nature and focused on known species distributions. A few references include Anthos (http://www.anthos.es/, accessed on January 2023), the Flora-On project (https://flora-on.pt/, accessed on January 2023), *Atlas de las plantas alóctonas de España* [32]. However, little information is available about the potential distribution and invasion risk of non-native plant species [33], specifically of non-native aquatic plant species. Such knowledge is available for certain established species [19,34] and for species with invasive potential [22]. When available, this information tends to be very specific and is not always easy to find. For instance, sometimes only a map of overall invasion risks is presented, which conveys information about all species without showing the potential distribution of target species independently. This study serves as an up-to-date and comprehensive source of information on the potential invasion risks of non-native aquatic plant species. It used the results of the Life Invasaqua project [23] to create a list of target species: non-native aquatic plant species currently established in the Iberian Peninsula as well as non-native aquatic plant species with invasive potential. In addition, the study’s findings were used to create the online Invasion Risk Atlas [35]. Easy to use, the atlas shows the potential distribution and, consequently, the areas at greatest risk of invasion by each of the target species. Both this study and the atlas represent a major advance with regards to existing resources for understanding the risk of invasion by non-native aquatic plant species in the Iberian Peninsula.

In the Invasion Risk Atlas [35], the maps for the different target species show variable macroecological patterns, which makes sense given the diversity in species types and ecologies (Figure 6). That said, many of the target species display similar patterns with regards to the nature of the areas at greatest risk of invasion. These are the areas that have the capacity to host a large number of non-native aquatic plant species, and they tend to occur near densely populated zones, coastal zones, and large river basins. These results concur with those obtained for other non-native aquatic species in the Iberian Peninsula, e.g., [36,37] and, specifically, for non-native aquatic plant species [19,20]. It is also important to note that zones, where non-native aquatic plant species richness is higher, tend to overlap with zones where native aquatic plant species richness is greatest, a key finding that should inform the conservation of aquatic biodiversity in the Iberian Peninsula [31]. This overlap likely stems from the fact that these areas are characterized by pronounced environmental heterogeneity. The factors that favor biological diversity occur in tandem with anthropogenic forces that facilitate the arrival and establishment of non-native species in communities of native species [38].

As highlighted in previous studies, climatic variables strongly influence potential species distributions [39,40], especially at the scales examined in this study and of ecological importance for aquatic plant species [19,20,31,36,41,42]. In the models, temperature—specifically the minimum temperature of the coldest month—was the variable of greatest importance. Its positive relationship with potential species richness and, consequently, invasion risk may be a consequence of how much temperature can limit species growth and development. In addition, the results showed that potential species richness was higher when the maximum temperature fell between 20 °C and 25 °C. Similar results have been obtained in previous research on non-native aquatic species [36,43] and on aquatic plant species in particular [20,42]. Additionally, precipitation, or the lack thereof, was also an important variable in the SDMs. First, it is an indicator of water availability and levels of soil moisture, both important abiotic variables in the life cycles of the target species. Notably, precipitation is key to species dispersion because it can create connections among water bodies. Second, precipitation levels serve as an indirect indicator of other conditions that could affect species distributions, such as modifications in the water regime or the concentration of nutrients [44,45,46]. In the case of the Iberian Peninsula, the areas at higher risk of invasion are those where rainfall is lower and displays greater seasonality, a pattern mentioned in past studies [19,20]. These finding underscores that climatically moderate zones may play an important role in facilitating invasions.

Consequently, the shifts in temperature and precipitation brought about by climate change could alter distribution patterns and the risk of invasion by non-native aquatic plant species. They could promote plant development and proliferation by creating new habitats with favorable conditions. Indeed, some currently non-problematic species could start to cause problems, move northwards, and even disappear entirely from their present ranges [42,43,47,48].

The use of non-climatic variables in SDMs can help improve predictions. Here, the inclusion of altitude yielded decent results in SDMs for certain species, such as *Spartina* spp. Schreb., *Halophila stipulacea* (Forssk.) Asch., and *Zostera japonica* Asch. & Graebn. (Figure 3 and Figure 4), which all occur in estuarine environments and marshes. This result is noteworthy because some have suggested that it is questionable to include this variable in SDMs for plant species [49]. Thus, it would appear to be important to account for altitude when modeling the distributions of aquatic plant species, especially those found in habitats where this variable matters, such as marshes and estuaries.

Although it is increasingly common for variables related to water chemistry to be included in SDMs, e.g., [41,50], their use is not always appropriate, as it will depend on the modeling scale. For example, water chemistry can help explain local species distribution patterns [40] but may not make the same contribution to higher-scale models, but see [41,51]. In contrast, variables that provide information about anthropogenic pressures, habitat accessibility, and propagule pressure have proven to be useful in SDMs for non-native species in general [36,39,52] and for non-native aquatic plant species in particular [19,20]. As a result, this study included the HFP index in its SDMs. Indeed, anthropogenic pressures can help explain the presence of certain non-native taxa in climatically unfavorable areas or their absence from climatically favorable areas: successful colonization relies at least in part on factors such as propagule pressure [52].

The results show that the HFP index and altitude present opposite trends in relation to potential species richness (Figure 7A,B). This pattern can be explained by the fact that, in the Iberian Peninsula, high-elevation areas are less accessible and, consequently, more pristine than lower-elevation areas, which experience greater anthropogenic pressure, propagule pressure, and nutrient availability. Other research has found similar results: non-native species are rare in or entirely absent from isolated areas but abundant in accessible areas [43]. In this context, it is worth noting that the richness of non-native species is directly correlated with the degree of anthropogenic activity within regions [36].

At the same time, limited and irregular levels of precipitation in low-lying areas may interact with high levels of anthropogenic activity to increase nutrient concentrations in watersheds [53], which can promote the arrival and establishment of certain non-native aquatic plant species. Such a relationship would support a hypothesis proposed elsewhere [19], which was based on the positive association observed between irrigated farmland and invasion risk in the Iberian Peninsula.

Based on the above, it seems likely that using variables that quantify the degree of anthropogenic pressure could have two important functions: to improve SDM quality, as also highlighted elsewhere [52], and to indirectly incorporate information normally contained in variables absent from the models, such as the level of nutrients in aquatic environments.

Both the established non-native species and the species with invasive potential overlapped in their areas at greatest risk of invasion. That said, there were subtle differences between the two groups (Figure 1B,C). The main reason may be that the models for established species contain data on actual species presence and environmental conditions in both the Iberian Peninsula and other invaded regions. Such is not the case for the species with invasive potential. Consequently, it seems important to include as much occurrence data as possible from a species’ native and invasive ranges when modeling potential distributions. Taking this approach will result in more robust models that account for the entire range of environmental conditions in which the species is found [47].

This study is not without its limitations. Sometimes the use of alternative settings is more appropriate for model development than the use of MaxEnt default options [54]. However, this would mean applying individual models for each species, which would go against standardizing and automating the process [55]. This is outside the scope of this work, as the original idea of this project is to implement a common protocol for all species studied, in order to save time and costs in all phases of control and management of non-native aquatic plant species. On the other hand, the lack of specific predictors based on the ecology of the species studied may be a limiting factor in this work [51]. However, as reflected in the manuscript and the references cited therein, the use of climatic variables and the impact of human activities on the area have worked well in the development of SDMs for non-native species [36,41]. It is possible that the use of general predictors may overestimate the potential distribution of the species studied, but it is preferable to overestimate rather than underestimate the potential distribution of species from the perspective of non-native species management [56]. It is important to remember that the resulting models are simplifications of reality, based on a small number of predictors that reflect habitat suitability rather than the absolute limits of species survival [56]. Despite the uncertainty of such spatial representations, SDMs are currently one of the most useful tools for detecting trends in the spread of non-native species.

This work is dynamic in nature and will evolve as new records on species presence are collected. In this way, the models will continue to improve in the future, as new non-native species are uncovered and studied by the scientific community. This characteristic is a strength, not a limitation. Indeed, the function of this article and the Invasion Risk Atlas [35] is to lay a foundation for developing targeted strategies and identifying new areas where non-native aquatic plant species occur. This information will help update the models and improve the resulting predictions.

## 4. Materials and Methods

### 4.1. Study Area

The focus of this study was the Iberian Peninsula, which comprises continental parts of Spain and Portugal. The Balearic Islands and the Macaronesian archipelagos were excluded, in accordance with recommendations made by the Life Invasaqua project [23]. Located in the Mediterranean Basin, the Iberian Peninsula is a hotspot of plant biodiversity [57,58]. It harbors a range of highly diverse native aquatic plant species [30,31] that are currently under threat given the arrival and presence of non-native aquatic plant species [19,20].

### 4.2. Species Occurrence Records

This study’s list of target non-native aquatic plant species was taken from the Life Invasaqua project [23] and was expanded using the project’s technical reports [59,60,61]. The list comprises 32 non-native aquatic plant species that have become established in the Iberian Peninsula (Table S1) as well as 41 non-native aquatic plant species with invasive potential (Table S2). The reference nomenclature was adopted from Tropicos (https://tropicos.org, accessed on August 2023).

Occurrence data for all 73 species were downloaded from GBIF (https://www.gbif.org/, accessed on January 2023; Table S3) and EASIN (https://easin.jrc.ec.europa.eu/easin, accessed on January 2023). A database was compiled after incorporating the global occurrence data (i.e., a species’ native and non-native distributions). Such an approach makes it possible to include the environmental breadth acquired by species in newly colonized areas, which reflects the species’ ability to acclimate [62]. It is therefore possible to produce more reliable maps than when using a more limited set of records [47].

Because data quality determines the model quality [63], a data cleaning protocol was applied. Erroneous data (geographical and taxonomic) were removed, as were data with a low degree of spatial resolution, i.e., zero coordinates and equal latitude and longitude data [64]. In addition, to reduce the influence of sampling bias in the models, duplicates were eliminated, and, to reduce spatial autocorrelation, the degree of data aggregation was reduced by retaining only one occurrence point per pixel with respect to the predictor variables grid [65,66]. Despite these efforts, it is possible that the data still displayed a degree of spatial autocorrelation. It may actually be a natural result of scattering processes and should not be artificially removed [67]. After the data cleaning process, the species records were ready for use in the SDMs and to establish macroecological patterns of species [55]. Species with less than ten records were excluded from the analyses [68].

All the analysis and management of the geographical data was performed using R v. 4.1.3 (https://www.r-project.org/ accessed on April 2023) and QGIS v. 3.28 (https://qgis.org/, accessed on May 2023). Furthermore, the QGIS PyQGIS library and Python (https://www.python.org/, accessed on May 2023) were used to automate repetitive processes.

### 4.3. Selection of Predictor Variables

The set of 19 bioclimatic predictor variables, which are described in Fick and Hijmans [69], and the digital elevation model (altitude) from the Shuttle Radar Topography Mission were downloaded from WorldClim (https://worldclim.org/, accessed on January 2023).

In addition, to include anthropogenic impacts, accessibility, and propagule pressure, values for the human footprint (HFP) index were downloaded from the Socioeconomic Data and Applications Center (https://sedac.ciesin.columbia.edu/, accessed on January 2023). This index includes eight variables that reflect regional anthropogenic pressures: human constructions, population density, electricity infrastructure, croplands, pastures, roads, railways, and waterways [70].

All the variable values were compiled and associated with the EPSG:3857 reference coordinates system (spatial resolution: 5 arc min). Given the large number of species being modeled, this level of resolution best balances computational space and time with model accuracy [36]. Variables were selected by determining their ecological significance for the target species, drawing on previous studies [41,42,47]. Next, to minimize model overfitting and optimize model parsimony, the number of variables was refined using the variance inflation factor (VIF). This factor expresses the degree of collinearity among variables; here, variables with a VIF value of greater than 5 were eliminated [11] using the R package HH [71]. The variables retained were the digital elevation model (altitude), the maximum temperature of the warmest month (Bio5), the minimum temperature of the coldest month (Bio6), the seasonality of precipitation (Bio15), the precipitation of the driest quarter (Bio17), and the human footprint (HFP) index.

### 4.4. Species Distribution Models

The SDMs were calibrated using MaxEnt v. 3.4.4 (maximum entropy modeling; https://biodiversityinformatics.amnh.org/open_source/maxent/, accessed on April 2023), an algorithm that combines machine learning with the maximum entropy principle. MaxEnt estimates the probability that a species will be present by determining the maximum entropy distribution based on known species occurrence [72]. MaxEnt was chosen because it can be employed with presence-only data, even if relatively few records are available, and because it is not strongly influenced by spatial sampling errors [54,63,73,74]. Furthermore, MaxEnt performs and predicts potential areas comparatively well against ensemble models [75], making it one of the most reliable techniques for modeling distributions of incomplete datasets [76].

The SDMs were carried out with MaxEnt using the default parameters (‘Auto features’, convergence = 10–5, the maximum number of iterations = 500, prevalence = 0.5, regularization value β = 1) [72,77,78], an approach previously used to model the potential distributions of aquatic plant species in general [31] and non-native aquatic plant species in particular [19,20]. As only presence data were used, 10,000 random background points were generated and employed as “pseudoabsences” when running the models. The predictive performance of each model was evaluated using a 10-fold cross-validation procedure, where 80% of the data were employed to train the model and the remaining 20% were utilized to validate the model. The predictive accuracy of the models was determined by analyzing the area under the ROC curve (AUC). Modeling results were considered to express plausible habitat occupation if the AUC was greater than 0.7 [79]. To obtain the most robust estimate of model predictive performance, the average of 10 replicates of each model was used to represent potential species distributions [80].

### 4.5. Online Invasion Risk Atlas

The resulting maps of invasion risk for each target species have been made available online, resulting in the first invasion risk atlas for the Iberian Peninsula ([35], https://InvasionRiskAtlas.github.io/, accessed on July 2023). The atlas was developed using HyperText Markup Language (HTML), Cascading Style Sheets (CSS), and JavaScript scripting language. In addition, the Bootstrap v. 5 framework (https://getbootstrap.com/, accessed on July 2023) was used to speed up the web development process, which was accomplished using the Visual Studio Code (https://code.visualstudio.com/, accessed on July 2023) editor. The atlas was designed in accordance with responsive web criteria, which means it can be optimally visualized regardless of the viewing device (i.e., cell phone, tablet, computer). The atlas’ source code has been deposited in a GitHub repository (https://github.com/InvasionRiskAtlas?tab=repositories, accessed on July 2023), which has been made public via GitHub Pages.

### 4.6. Potential Richness of Non-Native Aquatic Plant Species

The modeling results, which expressed habitat suitability on a continuous scale, were transformed into maps expressing habitat suitability on a binary scale (presence/absence). This process used the maximum training sensitivity and specificity threshold, which produced the most accurate predictions [81]. The zones of binary suitability were delineated for the Iberian Peninsula and then summed to produce a map of the potential richness of non-native aquatic plant species, which showed the number of species per pixel that were likely to encounter conditions allowing colonization. Consequently, these are the locations where the risk of invasion by non-native aquatic plant species is expected to be highest [20].

In addition, using the ggplot2 package in R [82], response curves were generated for the variables included in the SDMs, with the goal of analyzing the relationships between these variables and the potential richness of non-native species in the Iberian Peninsula. The curves were fitted using generalized additive models (GAMs) because GAMs can handle nonlinear relationships between response and predictor variables [36,83].

## 5. Conclusions

The SDM results have emphasized the importance of considering climatic and anthropogenic variables when exploring the potential distributions of non-native aquatic plant species, which aligns with the findings of previous research on non-native aquatic plant species [19,20]. From the modeling based on these variables, it is evident that the Iberian Peninsula displays ideal environmental conditions for maintaining established populations of non-native aquatic plant species and for hosting new species with invasive potential. It is hoped that the results here as well as the taxon-specific information in the online Invasion Risk Atlas [35] will become important resources that can guide decision-making by environmental managers. This work should help build biogeographical and macroecological knowledge of aquatic plant species, a taxonomic group whose distribution patterns have been poorly characterized. Furthermore, this study presents an objective methodology that can be used with other taxonomic groups, which contrasts with previous work in this domain. Another advantage is that disseminating information about the potential distributions of non-native species can benefit the general public because knowledge and understanding are the first steps along the path to effective management. In addition, the information provided could help spur citizen science initiatives, for example, and thus contribute to data-gathering efforts and further model improvement. Finally, it is important to develop monitoring programs so that the arrival of non-native aquatic plant species in the Iberian Peninsula can be detected as early as possible. It is also essential to implement or support eradication, control, or mitigation programs in areas where the presence of non-native species can present a risk to the diversity of native species.

## Data Availability

The data are available in the author’s personal GitHub repository (https://github.com/InvasionRiskAtlas?tab=repositories, accessed on July 2023).

## Supplementary Materials

The following supporting information can be downloaded at: https://www.mdpi.com/article/10.3390/plants12173069/s1, Table S1: Results from SDMs performed for non-native aquatic plant species that are already established in the Iberian Peninsula. Occurrences = sample size used to run the model at the global scale after cleaning up the original occurrence data. AUC = area under the ROC curve. SD = standard error. MaxTSS = maximum training sensitivity and specificity threshold; Table S2: Results from SDMs performed for non-native aquatic plant species that have the potential to invade the Iberian Peninsula. Occurrences = sample size used to run the model at the global scale after cleaning up the original occurrence data. AUC = area under the ROC curve. SD = standard error. MaxTSS = maximum training sensitivity and specificity threshold. Table S3: List of links to download species occurrence records from the GBIF platform.
