# Supplementary material for: Identifying and Managing Areas under Threat in the Iberian Peninsula: An Invasion Risk Atlas for Non-Native Aquatic Plant Species as a Potential Tool
Source: Plants (Basel). 2023 Aug 26;12(17):3069. doi: 10.3390/plants12173069 (PMC10490461; doi:10.3390/plants12173069)
Supplement: Supplementary file 1 [file plants-12-03069-s001.zip › plants-2559295-supplementary.pdf]

Table S1. Results from SDMs performed for non-native aquatic plant species that are already established in the Iberian Peninsula. Occurrences = sample size used to run the model at the global scale after cleaning up the original occurrence data. AUC = area under the ROC curve. SD = standard error. MaxTSS = maximum training sensitivity and specificity threshold.

Species	Occurrences	AUC	SD	MaxTSS
Alternanthera philoxeroides (Mart.) Griseb.	2,762	0.888	0.005	0.238
Azolla filiculoides Lam.	4,827	0.831	0.006	0.306
Bacopa monnieri (L.) Pennell	1,797	0.900	0.007	0.232
Crassula aquatica (L.) Schönland	1,022	0.933	0.008	0.221
Egeria densa Planch.	1,438	0.920	0.006	0.248
Eichhornia crassipes (Mart.) Solms	2,278	0.875	0.007	0.284
Elodea canadensis Michx.	15,072	0.702	0.005	0.371
Heteranthera limosa (Sw.) Willd.	508	0.938	0.011	0.197
Heteranthera reniformis Ruiz & Pav.	667	0.938	0.008	0.243
Heteranthera rotundifolia (Kunth) Griseb.	175	0.949	0.017	0.236
Hydrocotyle bonariensis Lam.	1,290	0.929	0.006	0.193
Hydrocotyle ranunculoides L. f.	2,354	0.893	0.006	0.241
Hydrocotyle verticillata Thunb.	1,088	0.918	0.008	0.237
Lagarosiphon major Moss ex Wager	1,811	0.926	0.004	0.159
Lemna minuta Kunth	4,123	0.857	0.005	0.295
Lemna valdiviana Phil	177	0.933	0.020	0.194
Limnobium laevigatum (Humb. & Bonpl. ex Willd.) Heine	201	0.954	0.016	0.256
Ludwigia grandiflora (Michx.) Greuter & Burdet	1,935	0.917	0.005	0.246
Ludwigia peploides subsp. *montevidensis* (Spreng.) P.H.Raven	1,390	0.930	0.005	0.165
Ludwigia repens J.R. Frost.	260	0.952	0.017	0.142
Myriophyllum aquaticum (vell.) Verdc.	4,605	0.839	0.005	0.273
Myriophyllum heterophyllum Michx.	694	0.954	0.006	0.202
Najas gracillima (A.Braun ex. Engelm.) Magnus	155	0.969	0.016	0.137
Najas graminea Delile	243	0.948	0.021	0.196
Nymphaea mexicana Zucc.	215	0.976	0.008	0.082
Pistia stratiotes L.	3,127	0.846	0.007	0.291
Rotala indica (Willd.) Koehne	498	0.965	0.007	0.137
Salvinia natans (L.) All.	1,825	0.910	0.006	0.256
Salvinia molesta D. S. Mitch.	867	0.931	0.008	0.209
Spartina alterniflora Loisel.	307	0.981	0.003	0.112
Spartina densiflora Brongn.	68	0.993	0.005	0.042
Spartina patens (Aiton) Muhl.	241	0.985	0.004	0.047

Table S2. Results from SDMs performed for non-native aquatic plant species that have the potential to invade the Iberian Peninsula. Occurrences = sample size used to run the model at the global scale after cleaning up the original occurrence data. AUC = area under the ROC curve. SD = standard error. MaxTSS = maximum training sensitivity and specificity threshold.

Species	Occurrences	AUC	SD	MaxTSS
Aponogeton distachyos L. f.	569	0.968	0.005	0.126
Azolla microphylla Kaulf.	89	0.921	0.035	0.286
Cabomba caroliniana A. Gray	736	0.951	0.006	0.180
Callitriche deflexa A. Braun ex. Hegelm.	22	0.978	0.028	0.086
Crassula helmsii (Kirk) Cockayne	3,235	0.883	0.005	0.248
Eichhornia diversifolia (Vahl) Urb.	129	0.943	0.015	0.287
Elodea callitrichoides (Rich.) Casp.	62	0.993	0.004	0.029
Elodea nuttallii (Planch.) H. St. John	6,212	0.814	0.005	0.375
Gymnocoronis spilanthoides (D.Don ex Hook. & Arn.) DC.	258	0.980	0.005	0.111
Halophila stipulacea (Forssk.) Asch.	58	0.984	0.010	0.153
Heteranthera zosterifolia Mart.	39	0.972	0.028	0.240
Hydrilla verticillata (L. f.) Royle	2,045	0.879	0.008	0.245
Hydrocotyle moschata G. Forst.	526	0.976	0.003	0.163
Hydrocotyle sibthorpioides Lam.	3,190	0.874	0.005	0.241
Hygrophila polysperma (Roxb.) T. Anderson	148	0.981	0.013	0.078
Landoltia punctata (G. Mey) Les & D.J. Crawford	585	0.951	0.008	0.131
Lemna aequinoctialis Welw. *	3	-	-	-
Lemna perpusilla Torr	275	0.971	0.012	0.140
Lemna turionifera Landolt	1,459	0.911	0.007	0.263
Ludwigia alternifolia L.	1,516	0.934	0.004	0.213
Murdannia keisak (Hassk.) Hand. -Mazz.	1,088	0.947	0.005	0.181
Myriophyllum verrucosum Lindl.	874	0.945	0.005	0.226
Najas guadalupensis (Spreng.) Magnus	746	0.914	0.012	0.277
Nelumbo nucifera Gaertn.	1,272	0.907	0.009	0.230
Nuphar advena (Aiton) W.T. Aiton	1,282	0.937	0.005	0.158
Nymphaea lotus L.	591	0.917	0.012	0.178
Orontium aquaticum L.	744	0.963	0.003	0.262
Ottelia alismoides (L.) Pers.	333	0.944	0.014	0.155
Pontederia cordata L.	5,828	0.810	0.006	0.312
Potamogeton epihydrus Raf.	951	0.936	0.008	0.277
Rotala ramosior (L.) Koehne	603	0.945	0.009	0.175
Rotala rotundifolia (Buch. -Ham ex. Roxb.) Koehne	286	0.966	0.012	0.135
Sagittaria graminea Michx.	724	0.953	0.006	0.179
Sagittaria platyphylla (Engelm.) J.G. Sm.	393	0.971	0.007	0.167
Sagittaria rigida Pursh	309	0.971	0.009	0.240
Salvinia auriculata Aubl.	351	0.948	0.011	0.250
Salvinia minima Baker	837	0.952	0.006	0.129
Saururus cernuus L.	2,983	0.887	0.005	0.235
Spartina anglica C. E. Hubb.	834	0.964	0.003	0.170
Vallisneria nana R. Br	191	0.960	0.012	0.247
Zostera japonica Asch. & Graebn.	62	0.986	0.016	0.056

* *Lemna aequinoctialis* was excluded from the analyses due to the small number of records.

Table S3. List of links to download species occurrence records from the GBIF platform.

Species	GBIF Download link
Alternanthera philoxeroides (Mart.) Griseb.	GBIF.org (26 January 2023) GBIF Occurrence Download https://doi.org/10.15468/dl.uq5bmg
Aponogeton distachyos L. f.	GBIF.org (27 January 2023) GBIF Occurrence Download https://doi.org/10.15468/dl.c586zy
Azolla filiculoides Lam.	GBIF.org (26 January 2023) GBIF Occurrence Download https://doi.org/10.15468/dl.6an8nt
Azolla microphylla Kaulf.	GBIF.org (27 January 2023) GBIF Occurrence Download https://doi.org/10.15468/dl.3grwv8
Bacopa monnieri (L.) Pennell	GBIF.org (26 January 2023) GBIF Occurrence Download https://doi.org/10.15468/dl.7zbrbd
Cabomba caroliniana A. Gray	GBIF.org (27 January 2023) GBIF Occurrence Download https://doi.org/10.15468/dl.f9hzn9
Callitriche deflexa A. Braun ex. Hegelm.	GBIF.org (27 January 2023) GBIF Occurrence Download https://doi.org/10.15468/dl.z4tms3
Crassula aquatica (L.) Schönland	GBIF.org (26 January 2023) GBIF Occurrence Download https://doi.org/10.15468/dl.7n7ykj
Crassula helmsii (Kirk) Cockayne	GBIF.org (27 January 2023) GBIF Occurrence Download https://doi.org/10.15468/dl.7nmwq6
Egeria densa Planch.	GBIF.org (26 January 2023) GBIF Occurrence Download https://doi.org/10.15468/dl.dspmvz
Eichhornia crassipes (Mart.) Solms	GBIF.org (26 January 2023) GBIF Occurrence Download https://doi.org/10.15468/dl.gny4wh
Eichhornia diversifolia (Vahl) Urb.	GBIF.org (27 January 2023) GBIF Occurrence Download https://doi.org/10.15468/dl.69j9ar
Elodea callitrichoides (Rich.) Casp.	GBIF.org (27 January 2023) GBIF Occurrence Download https://doi.org/10.15468/dl.h2ha2u
Elodea canadensis Michx.	GBIF.org (26 January 2023) GBIF Occurrence Download https://doi.org/10.15468/dl.j5kmk6
Elodea nuttallii (Planch.) H. St. John	GBIF.org (27 January 2023) GBIF Occurrence Download https://doi.org/10.15468/dl.mxebnd
Gymnocoronis spilanthoides (D.Don ex Hook. & Arn.) DC.	GBIF.org (27 January 2023) GBIF Occurrence Download https://doi.org/10.15468/dl.a4p5e9
Halophila stipulacea (Forssk.) Asch.	GBIF.org (27 January 2023) GBIF Occurrence Download https://doi.org/10.15468/dl.9cufux
Heteranthera limosa (Sw.) Willd.	GBIF.org (26 January 2023) GBIF Occurrence Download https://doi.org/10.15468/dl.nv3hrj
Heteranthera reniformis Ruiz & Pav.	GBIF.org (26 January 2023) GBIF Occurrence Download https://doi.org/10.15468/dl.fnnnvc
Heteranthera rotundifolia (Kunth) Griseb.	GBIF.org (26 January 2023) GBIF Occurrence Download https://doi.org/10.15468/dl.c58762
Heteranthera zosterifolia Mart.	GBIF.org (27 January 2023) GBIF Occurrence Download https://doi.org/10.15468/dl.q6vger
Hydrilla verticillata (L. f.) Royle	GBIF.org (27 January 2023) GBIF Occurrence Download https://doi.org/10.15468/dl.au64dd
Hydrocotyle bonariensis Lam.	GBIF.org (26 January 2023) GBIF Occurrence Download https://doi.org/10.15468/dl.uqhbm5
Hydrocotyle moschata G. Forst.	GBIF.org (27 January 2023) GBIF Occurrence Download https://doi.org/10.15468/dl.chtp36
Hydrocotyle ranunculoides L. f.	GBIF.org (26 January 2023) GBIF Occurrence

	Download https://doi.org/10.15468/dl.a4p5h5
Hydrocotyle sibthorpioides Lam.	GBIF.org (27 January 2023) GBIF Occurrence Download https://doi.org/10.15468/dl.m6vwex
Hydrocotyle verticillata Thunb.	GBIF.org (26 January 2023) GBIF Occurrence Download https://doi.org/10.15468/dl.sff9xz
Hygrophila polysperma (Roxb.) T. Anderson	GBIF.org (27 January 2023) GBIF Occurrence Download https://doi.org/10.15468/dl.4eq9np
Lagarosiphon major Moss ex Wager	GBIF.org (27 January 2023) GBIF Occurrence Download https://doi.org/10.15468/dl.u65z9u
Landoltia punctata (G. Mey) Les & D.J. Crawford	GBIF.org (27 January 2023) GBIF Occurrence Download https://doi.org/10.15468/dl.yzz7zu
Lemna aequinoctialis Welw.	GBIF.org (27 January 2023) GBIF Occurrence Download https://doi.org/10.15468/dl.xrhsu6
Lemna minuta Kunth	GBIF.org (27 January 2023) GBIF Occurrence Download https://doi.org/10.15468/dl.rkph69
Lemna perpusilla Torr.	GBIF.org (27 January 2023) GBIF Occurrence Download https://doi.org/10.15468/dl.586r66
Lemna turionifera Landolt	GBIF.org (27 January 2023) GBIF Occurrence Download https://doi.org/10.15468/dl.75jzg4
Lemna valdiviana Phil.	GBIF.org (27 January 2023) GBIF Occurrence Download https://doi.org/10.15468/dl.x6vevm
Limnobium laevigatum (Humb. & Bonpl. ex Willd.) Heine	GBIF.org (27 January 2023) GBIF Occurrence Download https://doi.org/10.15468/dl.atypv7
Ludwigia alternifolia L.	GBIF.org (27 January 2023) GBIF Occurrence Download https://doi.org/10.15468/dl.7qb4pq
Ludwigia grandiflora (Michx.) Greuter & Burdet	GBIF.org (27 January 2023) GBIF Occurrence Download https://doi.org/10.15468/dl.6x28x9
Ludwigia peploides subsp. *montevidensis* (Spreng.) P.H.Raven	GBIF.org (27 January 2023) GBIF Occurrence Download https://doi.org/10.15468/dl.ufhjz7
Ludwigia repens J.R. Frost.	GBIF.org (27 January 2023) GBIF Occurrence Download https://doi.org/10.15468/dl.t47ytc
Murdannia keisak (Hassk.) Hand.-Mazz.	GBIF.org (27 January 2023) GBIF Occurrence Download https://doi.org/10.15468/dl.h5uz6h
Myriophyllum aquaticum (Vell.) Verdc.	GBIF.org (27 January 2023) GBIF Occurrence Download https://doi.org/10.15468/dl.k7bxvj
Myriophyllum heterophyllum Michx.	GBIF.org (27 January 2023) GBIF Occurrence Download https://doi.org/10.15468/dl.sjmt6x
Myriophyllum verrucosum Lindl.	GBIF.org (27 January 2023) GBIF Occurrence Download https://doi.org/10.15468/dl.kye8jt
Najas gracillima (A.Braun ex. Engelm.) Magnus	GBIF.org (27 January 2023) GBIF Occurrence Download https://doi.org/10.15468/dl.tcr2pk
Najas graminea Delile	GBIF.org (27 January 2023) GBIF Occurrence Download https://doi.org/10.15468/dl.kz9ftm
Najas guadalupensis (Spreng.) Magnus	GBIF.org (27 January 2023) GBIF Occurrence Download https://doi.org/10.15468/dl.wbehhj
Nelumbo nucifera Gaertn.	GBIF.org (27 January 2023) GBIF Occurrence Download https://doi.org/10.15468/dl.dznqxm
Nuphar advena (Aiton) W.T. Aiton	GBIF.org (27 January 2023) GBIF Occurrence Download https://doi.org/10.15468/dl.dfhmkg
Nymphaea lotus L.	GBIF.org (27 January 2023) GBIF Occurrence Download https://doi.org/10.15468/dl.brk6hg
Nymphaea mexicana Zucc.	GBIF.org (27 January 2023) GBIF Occurrence Download https://doi.org/10.15468/dl.w4q8eb

Orontium aquaticum L.	GBIF.org (27 January 2023) GBIF Occurrence Download https://doi.org/10.15468/dl.kryx5b
Ottelia alismoides (L.) Pers.	GBIF.org (27 January 2023) GBIF Occurrence Download https://doi.org/10.15468/dl.ayzr5u
Pistia stratiotes L.	GBIF.org (27 January 2023) GBIF Occurrence Download https://doi.org/10.15468/dl.ux9wcp
Pontederia cordata L.	GBIF.org (27 January 2023) GBIF Occurrence Download https://doi.org/10.15468/dl.9nt6ap
Potamogeton epihydrus Raf.	GBIF.org (27 January 2023) GBIF Occurrence Download https://doi.org/10.15468/dl.t7d94c
Rotala indica (Willd.) Koehne	GBIF.org (27 January 2023) GBIF Occurrence Download https://doi.org/10.15468/dl.n5rc75
Rotala ramosior (L.) Koehne	GBIF.org (27 January 2023) GBIF Occurrence Download https://doi.org/10.15468/dl.9b9bpm
Rotala rotundifolia (Buch.-Ham. ex Roxb.) Koehne	GBIF.org (27 January 2023) GBIF Occurrence Download https://doi.org/10.15468/dl.wqtv48
Sagittaria graminea Michx.	GBIF.org (27 January 2023) GBIF Occurrence Download https://doi.org/10.15468/dl.s5g8fs
Sagittaria platyphylla (Engelm.) J.G. Sm.	GBIF.org (27 January 2023) GBIF Occurrence Download https://doi.org/10.15468/dl.ysfhmd
Sagittaria rigida Pursh	GBIF.org (27 January 2023) GBIF Occurrence Download https://doi.org/10.15468/dl.5cz6ac
Salvinia auriculata Aubl.	GBIF.org (27 January 2023) GBIF Occurrence Download https://doi.org/10.15468/dl.pkufxf
Salvinia minima Baker	GBIF.org (27 January 2023) GBIF Occurrence Download https://doi.org/10.15468/dl.7sh6jt
Salvinia natans (L.) All.	GBIF.org (27 January 2023) GBIF Occurrence Download https://doi.org/10.15468/dl.p59ymw
Salvinia molesta D. S. Mitch.	GBIF.org (27 January 2023) GBIF Occurrence Download https://doi.org/10.15468/dl.pjnasy
Saururus cernuus L.	GBIF.org (27 January 2023) GBIF Occurrence Download https://doi.org/10.15468/dl.makkbd
Spartina alterniflora Loisel.	GBIF.org (27 January 2023) GBIF Occurrence Download https://doi.org/10.15468/dl.jjtacg
Spartina anglica C. E. Hubb.	GBIF.org (27 January 2023) GBIF Occurrence Download https://doi.org/10.15468/dl.5k2z5n
Spartina densiflora Brongn.	GBIF.org (27 January 2023) GBIF Occurrence Download https://doi.org/10.15468/dl.873sxh
Spartina patens (Aiton) Muhl.	GBIF.org (27 January 2023) GBIF Occurrence Download https://doi.org/10.15468/dl.krdfy3
Vallisneria nana R. Br.	GBIF.org (27 January 2023) GBIF Occurrence Download https://doi.org/10.15468/dl.c9efjq
Zostera japonica Asch. & Graebn.	GBIF.org (27 January 2023) GBIF Occurrence Download https://doi.org/10.15468/dl.bybefz
